# Exploring Medical Professionals’ Perspectives on Cannabinoid Use in Professional and Elite-Level Contact Sports: A Qualitative Inquiry

**DOI:** 10.1177/19417381261490397

**Published:** 2026-10-12

**Authors:** Elizabeth S. Thompson, Jane Alcorn, Robert B. Laprairie, Noelle Rohatinsky, Payam Dehghani, J. Patrick Neary

**Affiliations:** † University of Saskatchewan, Saskatoon, Canada; ‡ University of Regina, Regina, Canada

**Keywords:** cannabis, cannabinoids, sports medicine, stigma, education, elite athletes, qualitative research

## Abstract

**Background:**

The legalization and growing use of cannabis in elite sport raise concerns about the preparedness of sports medical staff, who often lack formal cannabinoid education and face regulatory, ethical, and communication challenges.

**Hypothesis:**

Elite and professional contact sport medical staff have significant knowledge gaps, experience stigma, and face regulatory ambiguities that hinder open communication and athlete guidance on cannabis use.

**Study Design:**

Qualitative, descriptive study using thematic analysis of semi-structured interviews.

**Level of Evidence:**

Level 4.

**Methods:**

Semi-structured interviews were conducted with 10 medical staff (physicians, athletic trainers, nutritionists, and mental health professionals) from elite sports leagues in North America and Australia including the NHL, NFL, and NCAA. Interviews explored knowledge, experiences, barriers, and communication regarding cannabis. Transcripts were inductively coded to identify themes.

**Results:**

Six themes emerged: (1) pervasive “knowledge gap,” with most staff lacking formal cannabis education and relying on informal sources; (2) compliance challenges and regulatory uncertainty due to inconsistent policies; (3) stigma and fear limiting open dialogue; (4) shifting attitudes as societal norms evolve; (5) observed increase in athlete cannabis use and inquiries for recreational and therapeutic purposes, highlighting a knowledge-practice gap; and (6) trust and communication as prerequisites for honest discussion and safe guidance. These factors restrict access to informed, evidence-based advice.

**Conclusion:**

Elite sport medical professionals face educational and regulatory barriers, compounded by stigma and inconsistent policies. Despite evolving attitudes, most lack knowledge and institutional support to advise athletes, underscoring the need for education, research, and clear guidelines.

**Clinical Relevance:**

Targeted cannabinoid education, destigmatization, and clearer league policies are essential to optimize athlete care and harm reduction as cannabis use grows in elite sport.

Medical staff working with professional and elite-level athletes often lack education, experience, or quality resources related to cannabis and cannabinoid products and use.^8,9,26^ This knowledge gap is concerning, as cannabis legality and acceptance continue to grow in sports and society.^13^ This gap in knowledge can hinder both medical staff and athletes from making informed decisions about their health and performance, potentially exposing them to risks associated with unregulated cannabis products or misuse.^2^ Furthermore, without proper guidance, athletes may miss potential therapeutic benefits of cannabinoids that are being researched today.^28^

The absence of informed support or open communication from medical staff may lead to inconsistent practices, increasing the risk of misuse and negative outcomes for athlete well-being and performance.^3^ Historic legal status, such as removal from the United States pharmacopeia and subsequent criminalization, stifled research, education, and inclusion of cannabis content in healthcare training programs.^6^ Consequently, many clinicians are unfamiliar with cannabinoid mechanisms, potential benefits, and dosing protocols, adding risks for patients.^12^

With changing laws and increased interest in cannabinoid therapeutics, the lack of qualified cannabinoid specialists has allowed the profit-driven recreational commercial market to interject and fill this void.^15^ There is high demand for quality cannabinoid information among athletes and patients, yet practitioners are often ill-equipped to provide credible guidance due to limited education.^2,3,15,18^ As a result, many individuals now use cannabis therapeutically without direct healthcare provider input, diverging further from best practices.^2,3,18^

Elite athletes show particular interest in cannabinoid products.^7^ As leagues amend banned substance policies, medical staff assume increasing responsibility for guidance, even as many organizations maintain restrictive or inconsistent policies, creating discord in clinical management and compliance.^7,23-25^ Achieving improved athlete care and harm reduction as cannabis use grows in sport will require targeted education, destigmatization, and policy alignment to address persistent knowledge gaps, institutional uncertainty, and stigma among sports medicine professionals.^1,2,9,26^

## Objectives of the Study

The study aimed to: (1) describe the demographics of elite and professional contact sport medical staff regarding cannabinoid use; (2) describe the prevalence and reasons for use, educational background, and information sources; (3) assess the desire for further education and preferred educational formats; (4) outline experiences with stigma and access barriers to cannabinoid therapy; (5) characterize the communication and trust dynamics between elite athletes and their medical support staff; and (6) describe discrepancies in knowledge, opinions, and perspectives between athletes and medical staff regarding cannabinoid therapy. Achieving these objectives will guide the creation of strategies and educational resources to support medical professionals in providing informed cannabis advice and help athletes explore therapeutic cannabis use. The findings may drive further resource allocation and research on cannabis in professional sports.

## Methodology

### Design and Participants

Semi-structured interviews were conducted with 10 medical professionals from professional or college-level sports teams (NHL, NFL, NBA, MLB, AFL, NCAA, and USports), guaranteeing confidentiality. Participants, team doctors, trainers, nutritionists, and mental health professionals (men or women, age 25–55), were recruited via university/social media postings or personal invitation after survey completion. Due to the sensitive nature of cannabis use in professional sports, recruitment for this study was inherently challenging. Many medical and performance professionals working within elite sport organizations were hesitant to discuss cannabis-related practices or perspectives because of concerns related to professional reputation, organizational policies, and self-preservation, despite assurances of confidentiality and anonymity. In addition, gaining access to professionals within high-performance sport settings was difficult due to limited availability. Qualitative interviewing and thematic coding are highly time-intensive processes that require substantial depth of analysis for each participant, as such, a sample of 10 participants provided an appropriate and manageable starting point for obtaining initial insights while maintaining thorough qualitative analysis.

### Data Collection

A semi-structured interview format enabled in-depth discussion of knowledge, attitudes, personal experience, safety concerns, legal context, team rules, and communication dynamics. Interviews lasted 30–45 minutes, using a guide to ensure consistent topic coverage and allowing flexibility for open-ended insights.

### Procedure

All interviews were conducted by a single researcher (E.T) over Zoom video conferencing (Zoom Video Communications Inc. (2024). Zoom [Computer software] San Jose, CA), recorded with participant consent, and transcribed verbatim. Transcripts were imported into NVivo 14 software (Lumivero (2023). NVivo (Version 14) [Computer software] Denver, CO) for systematic data management and analysis.

### Data Analysis

Braun and Clarke’s thematic analysis was applied^5^: (1) familiarization with transcripts; (2) systematic inductive coding; (3) collating codes to themes; (4) theme review to ensure alignment with the dataset; (5) naming and definition of themes; and (6) producing a report linking findings to the research question with illustrative quotations.

### Researcher Reflexivity Statement

As the primary researcher with 25 years’ experience in professional sports and academic expertise in cannabinoids, E.T. recognizes background influences on her role in this study. While her knowledge helps contextualize medical staff experiences, she has deliberately separated it from participant perspectives to maintain objectivity. E.T. holds a neutral stance on cannabis, having witnessed both positive and negative effects, which informs an unbiased approach. Motivated to understand medical staff and athlete perspectives and improve education amid evolving cannabis laws and sports policies, E.T. employed thematic analysis with reflexivity to ensure transparency, identify potential biases, and uphold rigor throughout the research process.

## Results

The demographics of medical staff that were interviewed included men (n = 6) and women (n = 4), with an age range of 25 to 55 years. All medical staff had been practicing in their designations for at least 6 years, with several having over 15 years of experience. An approximately equal number of medical staff had never used cannabinoid products compared with those who had for either therapeutic or recreational purposes. All medical staff were in Canada, the United States, or Australia and included strength and conditioning coaches, physicians, team nutritionists, mental health specialists, and athletic therapists.

Six main themes were identified, with subthemes existing within most primary themes (Table 1). For a more expanded collection of quotes, please see Supplemental Material (available in the online version of this article).

**Table 1.** Primary themes and subthemes

Primary themes	Subthemes
The Green Knowledge Gap: The Break in Medical Expertise	• Not Educated About Cannabis • Education Sources • Credible Cannabis Education
Play By the Rules: The Influence of Compliance in Sports	• Rules Are Top Down • Prioritize Proper Compliance • Acceptance of Alcohol in Sport • Legalization Increased Interest
The Burden of Bias	• Fear
The Curtain is Rising	• Lived Experience and Societal Influences • A Better Alternative • Barriers to Perspective Shift
Athletes in the Green Zone: Navigating Cannabis for Remedy, Recreation, and Performance	• Recreational Use • Therapeutic Use • Athletes Learn About Cannabis
Connect and Convey: Communication Bridges All Knowledge Gaps	

## The Green Knowledge Gap: The Break in Medical Expertise

Medical staff in professional sports face significant barriers when addressing cannabis-related issues due to limited education and experience. Many express that “the education that I’ve received on it [cannabis] has been pretty much nothing, so anything helping to educate myself and the performance department on it would be beneficial” (MS10). This knowledge gap hinders their ability to advise athletes, as seen in three subthemes: Not Educated About Cannabis, Education Sources, and Credible Cannabis Education.

### Not Educated About Cannabis

Most staff reported feeling unqualified to answer questions about cannabis or provide guidance, often admitting, “I’ve had no formal training” (MS9). The lack of knowledge leads to discomfort, with one respondent explaining, “Feeling uneducated in something and not having an answer when an athlete comes to you is a bad feeling” (MS10). Even those with advanced degrees noticed gaps: “I have my master’s degree, and we didn’t touch it [cannabis education]. Never talked about it” (MS7).

### Education Sources

To fill this gap, staff often turn to informal sources, relying heavily on the internet or peers, though as 1 noted, “there isn’t a ton of information available” from professional organizations or workplaces (MS1). This leads to uncertainty about credibility: “Can I trust this [internet source]? . . . I would have no idea where to go [for quality cannabis education]” (MS2). Formal education and institutional support are cited as noticeably absent.

### Credible Cannabis Education

Staff voiced strong interest in credible, expert-led cannabis education “I think if there is a course that is provided, I would definitely love to sign up for that, just to get more knowledge” (MS5) and suggested education should address practical application relevant to athletics. The importance of credible instruction was summarized: “If you’re doing that work and you have the credibility of an institution; I think that is by far the most credible thing. . . in the cannabis industry” (MS6). Being better educated, they say, would help them “counsel and give perspective to athletes” (MS9).

## Play By the Rules: Navigating Regulation in Sports Medicine

Medical staff face significant uncertainty and hesitancy regarding substances such as cannabis due to diverse laws, league policies, and the need to strictly follow established rules. As one staff member summarized, “My goal as an employee is never to go against any of the regulating bodies, any of the policies, any of the legislation. I operate within the law. I don't get close to the edges.” (MS6)

### Rules are Top Down

Team and league protocols enforce strict compliance with top-down regulations, resulting in pressure on staff and athletes to conform. “At the end of the day, WADA are the world anti-doping agency so they’re kind of the overlying boss there,” clarified one medical professional (MS6), reflecting the broad influence of anti-doping bodies. Communication is key to avoid confusion, as inconsistent enforcement or lack of clarity can create conflict and uncertainty.

### Prioritize Proper Compliance

Medical professionals prioritize athlete safety and career security by rigorously following existing laws and policies, even when these are unclear or evolving. “They don’t want to do anything at all to jeopardize their career in any way, shape or form,” noted one respondent (MS4). Conflicting or vague federal, state, and league rules about cannabis use further hinder dialogue and trustworthy guidance. As a result, many staff avoid advising on cannabis altogether, underlining: “I would only talk about CBD for legal reasons because that way nobody could make a mistake. . .” (MS6).

### Acceptance of Alcohol in Sport

Alcohol remains widely accepted and normalized in sports culture, in contrast with the strict controls on cannabis. “You’re actually considered an outsider if you don’t do it [drink alcohol], or don’t participate,” said one staff member (MS10). Sponsorships and celebrations highlight the disparity: “Every team is sponsored by alcohol, yet as soon as you mention cannabis it’s a big no, no.” (MS4). Many recognize that, despite health risks, alcohol is encouraged while cannabis remains taboo.

### Legalization Increased Interest

Shifts in cannabis laws have fostered openness and curiosity. “I think seeing it [cannabis] be legalized. . . played a part in it [wanting to learn about cannabis],” observed one provider (MS10). Legalization encourages staff and athletes to discuss cannabis use more freely and increases inquiries, as staff feel more able to address the topic: “I am more open to have those conversations if they bring it up.” (MS5).

## The Burden of Bias

Negative perceptions and longstanding stigma continue to shape how cannabis use is viewed among athletes, medical staff, and society, making open discussion difficult. “How serious about being great at your sport are you if this [cannabis] is what you’re doing right?” (MS1) captures the automatic judgment about commitment when an athlete uses cannabis. Staff described athletes as aware of the negative perception: “I think they are aware that it [cannabis use] would be seen negatively, and that it would be seen that they’re not taking their time here seriously.” (MS1) Compliance testing adds to the stigma, leading some to see cannabis as “more negative than alcohol from an upper echelon level mostly because there’s a testing associated with it” (MS1).

Stigma is rooted in culture and education, reinforced since childhood. “Even when I was in middle school and high school, it [cannabis] was extremely bastardized and seen as a sin almost. . . to be around, be associated with anybody who even did marijuana” (MS10). Cannabis-related conversations are rare among professional peers: “We can talk about the benders [alcohol consumption] on the weekend. . . But it [cannabis] is not something, like a bong party. Like that stuff [cannabis] is not talked about, certainly not amongst professional adults in a professional field” (MS3). Though some medical staff notice stigma is “becoming a little less stigmatized, and guys are speaking a little bit more openly about it” (MS9), cannabis remains largely taboo, with open discussion still difficult between athletes and staff.

### Fear

Fear is a dominant theme: athletes and staff worry about being judged, punished, or jeopardizing their positions in sport. “I don’t like it [athletes not communicating about cannabis use], but I think it’s more so just that fear of being judged” (MS1). Some athletes fear negative labeling: “because they don’t want to be seen as this pot smoking athlete. . .” (MS1). Avoidance is common, even with doctors, due to concern over consequences: “They are so petrified to even mention it [cannabis] to their doctor because they’re worried about the ramifications” (MS4). This anxiety also affects how medical staff interact with athletes, as one noted, “I think the guys have a tough time talking to me about it [cannabis], because they’re not really sure what or how I’m gonna react” (MS2).

## The Curtain is Rising: Shifting Attitudes

Attitudes toward cannabis among athletes and medical staff are evolving, with personal experience and social trends driving more openness to cannabis as a therapy. Medical professionals, particularly younger or research-focused staff, are increasingly seeing cannabis as a safer or more effective option than alcohol, opioids, or other prescription drugs. Despite a shift toward acceptance, negative stereotypes, ethical concerns, and regulatory hurdles still shape opinions and practice.

### Lived Experiences and Societal Influences

Direct exposure to cannabis, seeing its effects on patients, and changes in legal status are leading to gradually more positive perceptions. “So that’s my exposure, it was very low until I became a professional in a state where it’s legal,” explained one provider (MS1). Others found that “ the more patients I saw, the more benefits I saw. . . and the no side effects that everyone got gave me more and more confidence in the medication [cannabis]” (MS4). Personal and professional curiosity about cannabis use, rather than policy changes, often spurs deeper learning.

Societal acceptance and athlete culture also play a role. “You just see it [cannabis], dispensaries everywhere. It’s just. . . more common,” said one professional (MS8). Age differences matter, as younger staff and athletes are generally more open-minded: “I can probably bet that a lot of our guys do not go to him to ask him about this [cannabis] because he would have an old school response. . . younger guys [medical staff] with a different outlook. . .” (MS10).

### A Better Alternative

Medical staff increasingly perceive cannabis as safer than opioids or certain prescription drugs, especially in sports. “I would think it [cannabis] is safer, I would 100% think it’s safer [than opioids]. Again, not because I have enough knowledge of cannabis, but I have enough knowledge of the dangers of opioids. . .” (MS2). Long-term medication risks, including opioid addiction, have made staff more open to alternatives: “I have had athletes. . . being prescribed oxies. . . and now they’re addicted. And so, you know I’ve worked with them to get them off opioids” (MS4). Despite these views, discussions of cannabis as an option remain limited. “That’s the gap, nobody’s giving them the option. . . it’s all pharmaceuticals” (MS3).

### Barriers to Perspective Shift

Progress is slowed by stubborn stereotypes, ethical skepticism, and regulatory gaps. “And still the public perception. . . is as soon as you mentioned cannabis, it’s a ‘drug’, you know. It’s a shame” (MS4). Historical influences, like the war on drugs, linger: “I grew up in the Reagan era and Nancy Reagan’s war on drugs made it sound like it [cannabis] was going to be the demise of our future” (MS3). Regulatory confusion, lack of certified products, commercial interests, and generational divides all impede full acceptance. One professional observed, “Unfortunately, some of the ‘shonky’ characters in the space have got authorized authority. . . pumping out high ridiculous levels of THC flower, which isn’t medicinal at all” (MS4).

## Athletes in the Green Zone: Cannabis for Remedy, Recreation, and Performance

This theme explores athletes’ growing use of cannabis, drawing on shifting perspectives and real-world experiences reported by medical staff. Athletes increasingly choose cannabis for both recreational and therapeutic reasons, often independent of medical approval. Three subthemes are addressed: Recreational Use, Therapeutic Use, and Athletes Learning About Cannabis.

### Recreational Use

Athletes frequently use cannabis for relaxation and enjoyment, reflecting changing social norms and the growing acceptance of cannabis in the sports community. “I do view it as something more recreational mostly because that’s how it’s being treated by the people I’m kind of working with,” reported one staff member (MS1). Some liken its acceptance to that of alcohol, noting, “I think I see it now a little bit more like alcohol. . . mostly because that’s how my athletes are discussing it with me” (MS1). Athletes prefer cannabis for perceived benefits over alcohol, such as fewer negative side effects and less disruption to training. “I definitely know of our athletes that use it recreationally, yeah” (MS10).

### Therapeutic Use

Cannabis use among athletes extends beyond recreation to address health needs. Medical staff report athletes seek cannabis to manage pain, inflammation, anxiety, and sleep problems. “There’s been some pretty positive benefits [from using cannabinoids], and. . . it’s been something that has helped them,” said one staff member (MS9). Athletes use cannabis as an alternative to traditional medications when other treatments are insufficient: “I am gonna use it because I’m in so much pain because the Advil. . . is not working” (MS5). Usage is often practical rather than recreational, “It’s not the act of getting high that they’re going after” (MS5).

### Athletes Learning About Cannabis

Athletes are increasingly initiating discussions with staff about cannabis, focusing on its therapeutic effects and compliance with league rules. “Several athletes have brought up using marijuana and asked for my perspective,” noted 1 medical professional (MS9). Concern remains over the reliability of athletes’ information sources, as staff believe athletes often rely on the internet or anecdotes: “They just hear it [cannabis information] from other professional sports, from other common media sources” (MS8). Staff also acknowledge their own knowledge gaps: “I was honest with them about not knowing that [information on cannabis]. . . well, there is no good answer” (MS1). Both athletes and staff express concern about the lack of trustworthy education, with athletes seeking support they often do not receive.

## Connect and Convey: Communication Bridges All Knowledge Gaps

This theme explores how trust, confidentiality, and open dialogue impact communication between athletes and medical staff about cannabis. Athletes are more likely to discuss cannabis with staff they find approachable, emphasizing the importance of a supportive, nonjudgmental environment. Confidentiality is vital, as fear and stigma continue to make open communication challenging.

Limited practical experience with cannabis persists in the medical community, with many staff avoiding the topic: “It [cannabis] is, I would say, not openly discussed, still pretty taboo” (MS1). Leadership is needed to promote open discussion: “It really needs to come down from the top. . .opening up that dialogue. . .and trying to find more information for our people” (MS3).

Trust is foundational for athletes to share openly about cannabis. “It’s nice to have that open line of communication just for them to know that they can trust us” (MS5). Building trust can take time, but it leads to honest conversation: “So a lot of times it takes at least a full season to really build trust and relationships where the guys can use the space and have faith in it” (MS9). Respecting athlete agency and reassuring confidentiality encourages disclosure: “I think guys are more prone to discuss it with me because they know that I won’t put them on the drug test list” (MS1).

Athletes remain hesitant at times, concerned about how their information might be used, especially when staff are directly affiliated with organizations: “There’s some hesitation or fear because I’m contracted through the organization that they don’t know where that information is going” (MS9). Medical staff often take a reactive approach: “I am more open to have those conversations if they bring it [cannabis] up. . . I am more open to discussing it. . .versus. . .‘oh hey, are you smoking weed to get rid of your pain?’” (MS5).

Open communication and collaboration among medical staff, while valuing athlete participation in treatment decisions, are seen as best practice: “The [treatment] decision is not coming from one practitioner, and that’s a collaborative approach that creates a more holistic process.” (MS8). Ongoing education for organizations, families, and athletes is encouraged: “I think that educating everyone, all the sporting bodies, the people in charge, coaches, parents, I think only good can come from it” (MS7).

## Discussion

This qualitative study explored the knowledge, opinions, and perspectives of 10 individual medical staff members working in elite professional and collegiate contact sports regarding cannabis use. It is intended as a preliminary exploratory investigation into an understudied and sensitive topic within professional sport. To the best of the authors’ knowledge, this is the first qualitative investigation using professional and elite sport team doctors and training staff to examine healthcare providers’ perspectives on cannabis use in elite athletic settings. As such, the purpose of this study was not to provide definitive or fully generalizable conclusions across all professional roles, but rather to generate initial insight into themes and perspectives that may exist within this population and to help inform future research directions. Through thematic analysis of semi-structured interviews, several key themes were identified which provide insight into the complex landscape of cannabis in professional and elite athletics.

A prominent finding was the significant knowledge gap among athletic medical staff regarding cannabis and its potential therapeutic applications in sports. Most participants expressed a lack of formal training and education on cannabis, echoing findings from previous studies that have documented similar deficits among healthcare professionals.^8,10,22,28,30^ This deficiency impedes the ability of staff to advise athletes and creates barriers to informed clinical decision-making. The demand for expert-led, evidence-based educational initiatives to close this gap was strongly endorsed.^8,10,22,28,30^

Medical staff described ongoing confusion and anxiety about legal status, league policies, and compliance requirements related to cannabinoids. These ambiguities discourage open communication and appropriate athlete support, aligning with research highlighting the need for harmonized, clear policy for cannabis use in sport.^11,16^ Legal inconsistencies also drive aversion to risk, leading medical staff to restrict advice in order to avoid possible career repercussions.^11,16^

Deep-seated stigma and negative social perceptions continue to shape the attitudes of clinicians toward cannabis. Anti-drug campaigns and institutional messaging have historically contributed to silence and apprehension in discussing cannabis use, limiting harm reduction strategies.^12,14^ However, societal trends and legalization efforts have begun to reduce stigma, with younger and research-oriented medical staff adopting more open, health-centered perspectives.^17,18^

Medical staff acknowledge athletes are increasingly self-medicating with cannabinoids for pain management, recovery, and mental health, often in the absence of formal medical guidance. These trends underscore the risk of misinformation and unsafe practices but also reflect the necessity for open, nonjudgmental clinical relationships.^4,15,26^ Building trust and establishing confidential, evidence-based dialogue were identified as critical for effective athlete care. Policy clarity, team leadership, and stigma reduction efforts are essential to ensure athletes can seek guidance without fear of repercussion or judgment.^1,6,18,20^

In summary, this study supports existing literature calling for comprehensive medical education, regulatory reform, and proactive team communication to safely manage growing cannabinoid use in professional and elite contact sport environments.^4,7,8,10,22,26,30^

## Implications for Practice and Policy

The findings of this study have several implications for sports medicine practice and policy. Although limited sample size and professional heterogeneity may restrict the transferability of findings to all sports and medical professions, recurring patterns and thematic repetition were observed across interviews, suggesting preliminary thematic sufficiency within the scope of this exploratory study.

Improvements in cannabis education and training programs tailored to sports medicine professionals are needed to address the significant knowledge gap highlighted by the medical staff interviewed.^12,29^ Second, sports organizations should consider developing clearer guidelines and communication strategies regarding cannabis use to help medical staff navigate regulatory complexities. Third, efforts to destigmatize cannabis use and promote open dialogue between medical staff and athletes are necessary to ensure proper care and harm reduction. Fourth, research is needed to evaluate the potential benefits and risks of cannabis use in athlete populations, particularly as an alternative to traditional pain management approaches.^4,19,21,28^ Finally, sports organizations should assure alignment of policies regarding cannabis use in sports with the evolving scientific evidence and societal attitudes.^14,27,28^

## Limitations and Future Directions

This study is limited by its qualitative design and relatively small sample size, reflecting the perspectives of medical staff from a select group of elite professional and collegiate contact sports. Recruiting participants from professional sport organizations for discussions surrounding cannabis-related practices proved challenging due to the sensitive nature of the topic and concerns surrounding professional reputation and confidentiality. With future changes in Schedule 1 and reclassification of marijuana will open up the opportunity for medical professionals to speak openly about this topic.

Results may not generalize to all sporting contexts, geographic regions, or medical specialties. The self-reported nature of interviews may also introduce bias or omit perspectives from less-represented staff roles or organizations. Additional limitations include potential interviewer effects and the evolving regulatory landscape during the study period, which may influence staff attitudes and responses. The findings and recommendations should be interpreted as exploratory and hypothesis-generating rather than conclusive, with future studies employing larger and more profession-specific samples to further validate and expand upon these initial observations.

Future research could utilize larger, more diverse samples across multiple sports and geographic regions to assess generalizability and regional variations in cannabinoid management. Quantitative investigations and longitudinal studies can better elucidate the clinical outcomes and health effects of cannabinoid use for athletes. Interventional research into targeted education, team policy, and stigma reduction strategies is needed to establish best practices and support evidence-based care. Collaboration between sports organizations, clinical educators, and regulatory bodies will be essential to optimize athlete safety and harmonize approaches to cannabis in professional sport.

## Conclusion

This study has highlighted the complex and evolving landscape of cannabis use in professional and elite sports from the perspective of medical staff. The findings identify a considerable lack of knowledge about cannabis use by medical personnel working with athletes in elite level sports and underscore the pressing need for targeted education and policy alignment. The persistence of stigma, regulatory ambiguity, and variable clinical guidance emphasizes the importance of future research and leadership in shaping safe, effective athlete management for cannabinoid use.^15,17,28^

## Supplemental Material

sj-docx-1-sph-10.1177_19417381261490397 – Supplemental material for Exploring Medical Professionals’ Perspectives on Cannabinoid Use in Professional and Elite-Level Contact Sports: A Qualitative Inquiry Supplemental material, sj-docx-1-sph-10.1177_19417381261490397 for Exploring Medical Professionals’ Perspectives on Cannabinoid Use in Professional and Elite-Level Contact Sports: A Qualitative Inquiry by Elizabeth S. Thompson, Jane Alcorn, Robert B. Laprairie, Noelle Rohatinsky, Payam Dehghani and J. Patrick Neary in Sports Health
