# Supplementary material for: Exploring Medical Professionals’ Perspectives on Cannabinoid Use in Professional and Elite-Level Contact Sports: A Qualitative Inquiry
Source: Sports Health. 2026 Oct 12:19417381261490397. Online ahead of print. doi: 10.1177/19417381261490397 (PMC13658636; doi:10.1177/19417381261490397)
Supplement: Supplementary file 1 [file sj-docx-1-sph-10.1177_19417381261490397.docx]

**Supplemental Information: Participant Quotes by Theme**

These additional quotations expand on themes discussed in the main Results. They are provided here in full to support transparency and enable readers to appreciate the range and nuance of participant perspectives.

**The Green Knowledge Gap: The Break in Medical Expertise**

"Feeling uneducated in something and not having an answer when an athlete comes to you is a bad feeling." (MS10)

"I’ve had no formal training." (MS9)

"Honestly, I have no training whatsoever." (MS5)

"I’ve had zero training." (MS8)

"I don’t think we know what questions to ask, and so we haven’t been the best at educating ourselves on what we need to know." (MS1)

"Most of my training [on cannabis] has come from online." (MS4)

"Can I trust this [internet source]? You gotta vet it ... I would have no idea where to go [for quality cannabis education].” (MS2)

"If I could help teach them or show them a proper way to use it rather than them just find a few things off the shelf and trying it willy-nilly would be very beneficial." (MS10)

**Play By the Rules: The Influence of Compliance in Sports**

"I can’t make a recommendation [about cannabis] directly to the athlete, that’s outside my scope of practice." (MS6)

"They don’t want to do anything at all to jeopardize their career in any way, shape or form." (MS4)

"It’s not openly discussed. It’s still pretty taboo." (MS1)

"I would only talk about CBD for legal reasons because that way nobody could make a mistake and say, oh well, coach told me I can do this, or I can do that, or this is okay." (MS6)

"You’re actually considered an outsider if you don’t do it [drink alcohol] or don’t participate." (MS10)

"I think alcohol is used more as a reward, like after the end of a long game, whatever it may be, you drink alcohol." (MS5)

**Stigma and Shifting Attitudes**

"Even when I was in middle school and high school, it [cannabis] was extremely bastardized and seen as a sin almost, to utilize, use, be around, be associated with anybody who even did marijuana." (MS10)

"Going through the D.A.R.E program ... all they talked about was, don’t drink, don’t smoke, don’t have sex, and without any real cause or effect as to why. It was just, don’t." (MS3)

"For some reason, cannabis is this weird sort of system at the moment where it’s not being treated like a medicine." (MS4)

"I think that’s the big thing, that it doesn’t matter what realm of sport, recreation, Joe public, or medical, the stigma is through and through all of those facets." (MS3)

"They [athletes] don’t directly bring it [cannabis] up to me, and to a lot of people it’s still very taboo." (MS2)

**Athletes in the Green Zone: Navigating Cannabis for Remedy, Recreation and Performance**

"That [cannabis information] tends to be anecdotal and is generally where they’re getting their information from, is other people’s anecdotes." (MS1)

"They see the benefit [of cannabis] but they don’t know the best course of action, or what is allowed and what is not allowed really to go forward from there." (MS8)

"Well, if I were to smoke or use an edible, or have any, or use weed I guess, how much can I use? Do you know what that threshold is? How do I stay under the threshold?" (MS1)

**Connect & Convey: Communication Bridges All Knowledge Gaps**

"So I think the guys have a tough time talking to me about it [cannabis], because they’re not really sure what or how I’m gonna react." (MS2)

"If you’re using this and self-medicating, we want to be aware of that and if you are abusing it, we wanna make sure you have resources for it." (MS1)

"I do try and keep that, be cognizant and pay attention to their conversations around it [ cannabis]. Pay attention to how they’re speaking about it." (MS1)

"I think that educating everyone, all the sporting bodies, the people in charge, coaches, parents, I think only good can come from it." (MS7)
